# Simple and robust 3D bioprinting of full-thickness human skin tissue

**DOI:** 10.1080/21655979.2022.2063651

**Published:** 2022-04-12

**Authors:** Jing Liu, Zhengtong Zhou, Min Zhang, Feng Song, Chong Feng, Haochen Liu

**Affiliations:** a School of Biology, Food and Environment, Hefei University, Hefei, China; b Department of Vascular Surgery, Xuanwu Hospital and Institute of Vascular Surgery, Capital Medical University, Beijing, China; c Shandong Key Laboratory of Biophysics, Institute of Biophysics, Dezhou University, Dezhou, Shandong, China; d Department of Cardiovascular Surgery, Xi’an Children’s Hospital, Xi’an, China

**Keywords:** Full-thickness skin, 3D bioprinting, bio-inks, stratum corneum formation

## Abstract

Artificial skins have been used as skin substitutes for wound healing in the clinic, and as in vitro models for safety assessment in cosmetic and pharmaceutical industries. The three-dimensional (3D) bioprinting technique provides a promising strategy in the fabrication of artificial skins. Despite the technological advances, many challenges remain to be conquered, such as the complicated preparation conditions for bio-printed skin and the unavailability of stability and robustness of skin bioprinting. Here, we formulated a novel bio-ink composed of gelatin, sodium alginate and fibrinogen. By optimizing the ratio of components in the bio-ink, the design of the 3D model and the printing conditions, a fibroblasts-containing dermal layer construct was firstly fabricated, on the top of which laminin and keratinocytes were sequentially placed. Through air-liquid interface (ALI) culture by virtue of sterile wire mesh, a full-thickness skin tissue was thus prepared. HE and immunofluorescence staining showed that the bio-printed skin was not only morphologically representative of the human skin, but also expressed the specific markers related to epidermal differentiation and stratum corneum formation. The presented easy and robust preparation of full-thickness skin constructs provides a powerful tool for the establishment of artificial skins, holding critical academic significance and application value.

## Graphical Abstract

**Figure:**
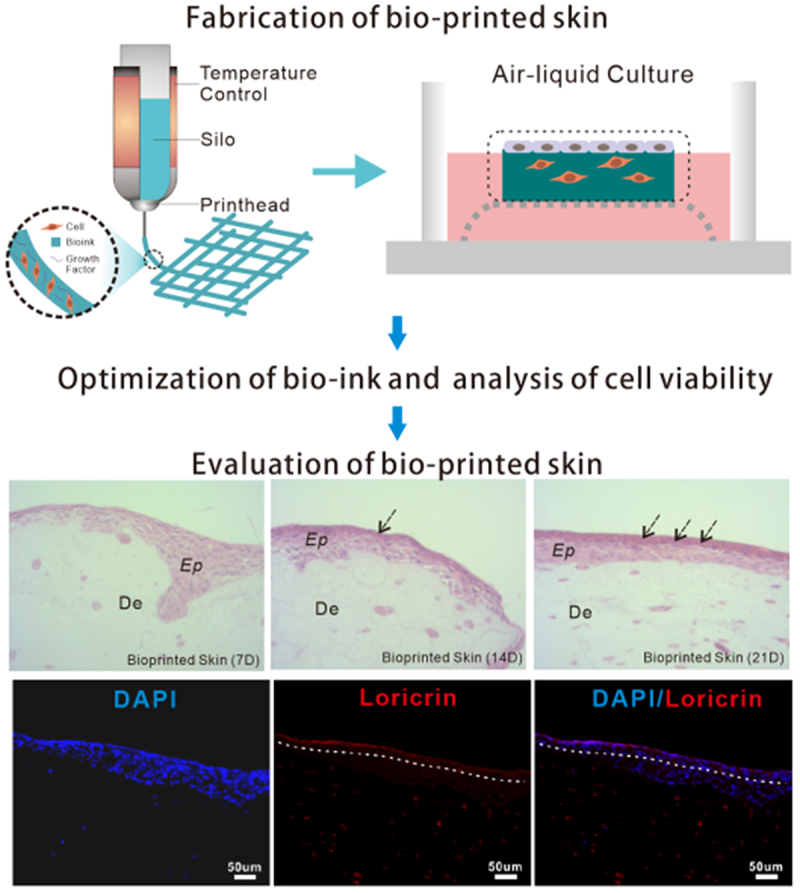

## 1. Introduction

Skin is the largest organ of the human body and plays a vital role in protecting the human body from the external environment [1,2]. Since skin is the body’s first line of defense, it is vulnerable to various damages such as traumatic wounds, burns, and diabetic feet [3,4]. When the skin is severely damaged and fails to heal itself, the main treatment currently is autologous grafts or tissue-engineered skin replacements [5]. Due to the limited transplantable autologous skin, it is particularly crucial to develop tissue-engineered skin substitutes for the treatment of skin damages [1,6]. In addition, European legislation has prohibited the use of animals for testing cosmetic ingredients, and meanwhile increased the number of industrial chemicals that must undergo risk assessment [7,8]. Therefore, there is an urgent need for high-quality skin substitutes to replace animals for testing in the fields of cosmetics and pharmacology.

3D bioprinting is a state-of-the-art technology to fabricate biological constructs with hierarchical architecture similar to their native counterparts by precisely locating living cells, extracellular matrix (ECM) components and various signaling molecules like growth factors in a layer-by-layer assembly [9,10]. Developing living functional tissues by artificial means can address unmet needs in tissue replacement, organ transplantation, and sample testing [11–13]. Thus, the 3D bioprinting technique provides a promising strategy in the fabrication of artificial skin or skin equivalents. Several researchers have devoted themselves to fabricating skin equivalents via 3D bioprinting. Vivian Lee et al. constructed a multi-layered cell and matrix structure to reproduce key morphological and biological features of in vivo human skin [14]; however, using collagen as the bio-ink resulted in poor printing accuracy. Prasad Admane et al. utilized the silk-gelatin bio-ink-based 3D bioprinting strategy to recapitulate a number of design and biological parameters akin to human skin [15], but the prepared skin failed to form a dense and thick epidermal layer. In addition, although Cubo N et al. used a fibrin-based bio-ink for the printing of primary human cells in form of sheets [16], the undulated pattern of the dermal-epidermal junction of the human skin was not demonstrated.

**Scheme 1.**
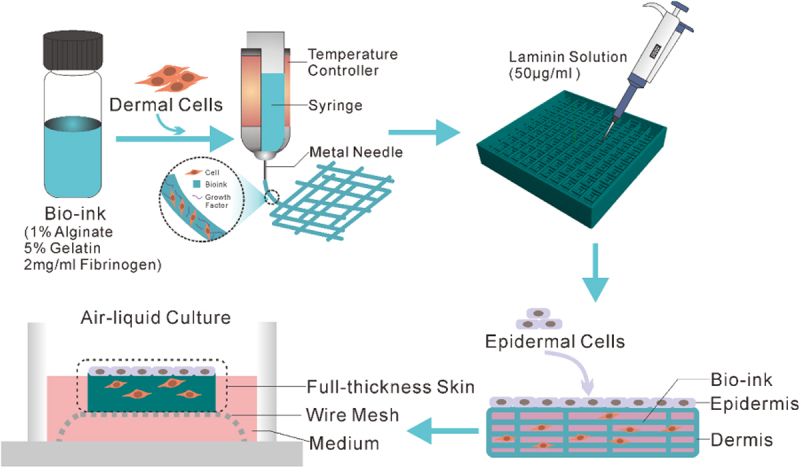
Schematic of fabrication of full-thickness human skin.

In this paper, we presented an approach that enables extrusion-based 3D bioprinting of a full-thickness human skin model in a simple, economic and robust way. Firstly, the skin frame was printed with bio-ink containing fibroblasts. Then, the thus-obtained skin frame was covered with the 50 μg/ml laminin solution followed by seeding of epidermal cells. Lastly, the full-thickness skin tissue was fabricated through ALI culture via wire mesh (Scheme 1). According to our experimental procedure, the printed full-thickness human skin will form a thicker epidermis and a distinct stratum corneum will be differentiated after 2 weeks of culture. Such a full-thickness human skin construct will prove to be highly applicable for drug or cosmetic testing in comparison to conventional trans-well cultures and animal models.

## 2. Materials and methods

### 2.1 Cell culture and expansion[17]

Human fibroblasts were isolated from human foreskin specimens (12 years old) obtained during circumcision surgery performed at the Department of Urology of Anhui Provincial Hospital. Informed consent was obtained from the patient. The isolation method can be summarized as follows: foreskin specimens were rinsed with PBS supplemented with 1% penicillin-streptomycin three times to remove the subcutaneous connective tissue and then spread in a flat dish. After the addition of Trypsin-EDTA (0.25%), the mixture was incubated overnight at 4°C to separate the epidermis and dermis. The dermis was cut into small pieces in a 35 mm petri dish, treated with 0.35% (w/v) Dispase II, and incubated for 60 min at 37°C. After being subjected to filtration and centrifugation, the primary fibroblasts were cultured in DMEM medium (Gibco) supplemented with 10% fetal bovine serum (Lonsera) and 1% penicillin-streptomycin (Gibco) at 37°C and 5% CO_2_ before being used within passage 4–12. Epidermal cells (HaCAT, human immortalized epidermal keratinocyte cell line) were purchased from ATCC and maintained in the same medium as that for primary dermal cells.

### 2.2 Preparation of bio-link

Bio-ink used in this study was formulated as a mixture of 5% (w/v) gelatin (Sigma-Aldrich), 1% alginate (Sigma-Aldrich), and 2 mg/ml fibrinogen (Sigma-Aldrich). Briefly, gelatin and alginate were dissolved at the required concentration in DMEM solution, and then stored for 1 hour at 60°C, followed by the addition of fibrinogen at the desired concentration. The thus-obtained mixture was then kept at 37°C. Fibroblasts were trypsinized and seeded in the bio-ink to obtain 2 × 10^6^ cells/ml in bio-ink, homogenized, and loaded in a sterile 10 mL cartridge. After eliminating air bubbles, the cartridge was stored in the bio-printer for at least 30 min at 10°C to make the ink printable.

### 2.3 3D model design

The digital 3D model was generated using BIOCAD software to simulate the dermal layer of human skin. The structure was designed with overall dimensions of 16 × 16 x 3 mm. The dermal layer consisted of a total of 10 layers. For the bottom 2 layers, the interfilament distance was kept at 0 mm to prevent cell suspension leakage. For the middle 4 layers and the top 4 layers, the interfilament distances were kept at 0.8 mm and 1.6 mm, respectively.

### 2.4 3D Bioprinting of dermal layer constructs

Bio-printing was carried out using the 3D Discovery bioprinter (Regenhu, Switzerland). The barrel temperature was maintained at 10°C using a temperature controller and the printing chamber temperature was maintained at 20°C. The bio-ink was dispensed through a sterile metal needle (nozzle diameter 0.33 mm), using a pneumatic pressure of 1.8–2.5 Mpa and a deposition speed of 5 mm/s. The deposition speed and pressure were controlled by customized software developed by Regenhu. After the dermal layer constructs were printed, they were crosslinked by sterile 2% (w/v) CaCl_2_. The crosslinked dermal constructs were washed with sterile saline solution thrice and dipped in DMEM (supplemented with 10% FBS, 1% penicillin-streptomycin) and incubated at 37°C and 5% CO_2_ .

### 2.5 Preparation of full-thickness skin constructs

After dermal constructs were successfully obtained, each of the dermal constructs was covered with 50 μg/ml laminin solution. Subsequently, epidermal cells were trypsinized, centrifuged, counted, and then evenly distributed on the prepared dermal constructs. Finally, the full-thickness skin models were transferred to sterile wire mesh for air-liquid interface culture with the DMEM differentiation medium supplemented with Human Keratinocyte Growth Supplement (HKGS, ThermoFisher), 50 μg/ml ascorbic acid, and 1.3 mM CaCl_2_ for 4, 7 and 21 days, and then were harvested for further analysis.

### 2.6 Cell viability assay

Cell viability of the printed constructs was conducted using Calcein‐AM and PI on day 7. In the experiments, the working concentrations of PI and calcein‐AM were 10 μg/ml and 5 μM, respectively [17]. The printed constructs were washed with PBS, incubated with the two dyes for 30 min at 37°C and then visualized by a high-content analysis system (CQ1, YOKOGAWA). In these assays, cells stained green/red were live/dead.

### 2.7 Histological analysis[17]

Full-thickness skins were collected after 7, 14, and 21 days, washed immediately with phosphate‐buffered saline, fixed with 4% formaldehyde at 4°C for 24 hr, and finally dehydrated in 70% ethanol. Thereafter, the samples were embedded in paraffin, sectioned serially, and stained with hematoxylin and eosin kit (Sangon, China).

### 2.8 Immunohistochemistry (IHC)

IHC was carried out to evaluate epidermal differentiation in the 3D bio-printed constructs using anti-loricrin antibody (Abcam, dilution 1:200,) and anti-cytokeratin 5 antibody (Abcam, dilution1:200) referring to the previous protocol [18]. Briefly, paraffin-embedded samples were cut into 7 μm sections. After dewaxing and rehydration, samples were incubated overnight with the primary antibodies at 4°C. Following this, secondary Alexa Fluor 488 and Alexa Fluor 594-conjugated anti-mouse or anti-rabbit antibodies were added and incubated for 1 h at room temperature. Nuclear counterstaining using DAPI (Sangon Biotech, China) was carried out routinely. Images were acquired using CQ1 (YOKOGAWA, Tokyo) high-content microscope.

### 2.9 Statistical analysis

All data were presented as mean ± standard deviation (SD) and subjected to one-way analysis of variance (ANOVA) followed by Bonferroni’s multiple-comparison tests with n as the number of different experiments.

## 3. Results

### 3.1 Formulation of bio-ink

In the skin 3D bioprinting process, the selection of bio-ink is of great importance in forming a stable skin construct. To prepare the bio-ink suitable for the extrusion-based bioprinting of skin tissue, we tested diverse combinations of biomaterials. Eventually, the bio-ink used in this particular study was optimized based on commonly used alginate/gelatin ink [19]. Huayu Yang et al. have tested the bio-ink with various proportions of gelatin and alginate, and found that gelatin at low concentrations (3% and 5%) in the bio-ink did not demonstrate a significant difference in cell viability, whereas cell viability decreased when the bio-ink contained 7% gelatin [20]. In view of this, and taking into account the structural formation, cell viability, as well as cell proliferation in prolonged culture, we first tested multiple combinations of gelatin and alginate in varying proportions, and ultimately used 5% gelatin and 1% alginate as a major component of bio-ink.

**Figure 1.**
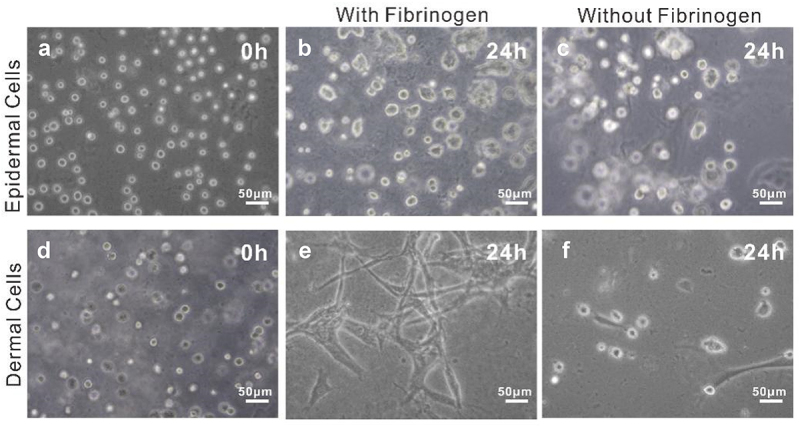
Optimization of bio-ink. A: Epidermal cells cultured in bio-ink at 0 h (control); B-C: Epidermal cells cultured in bio-ink with and without fibrinogen at 24 h; D: Dermal Cells cultured in bio-ink at 0 h (control); E-F: Dermal Cells cultured in bio-ink with and without fibrinogen at 24 h.

In addition, considering that fibrinogen is rich in several cell-binding sequences (such as Arg-Gly-Asp (RGD)) which are in favor of cell adhesion, proliferation, and differentiation [21,22], we optimized the bio-ink by adding fibrinogen as a component. To verify the performance of the bio-ink, we compared the cell morphology in bio-inks with and without fibrinogen. The results showed that after being cultured for 24 h, dermal cells were spindle-shaped in bio-ink with fibrinogen, whereas almost no spindle-shaped cells were observed in the bio-ink without fibrinogen (Figure 1Eand f). By comparison, no significant difference occurred to epidermal cells in the two bio-inks, which might be related to the morphology of the epidermal cells (Figure 1band c). In a word, the results revealed that bio-ink containing fibrinogen was more conducive to cell adhesion compared to the one without fibrinogen. In light of such results, the bio-ink consisting of 5% (w/v) gelatin, 1% alginate, and 2 mg/ml fibrinogen was chosen for subsequent bioprinting experiments.

### 3.2 Optimization of printing conditions

To apply bio-printed cellular constructs for skin tissue fabrication, it is imperative to guarantee the long-term survival and maintenance of functional cells encapsulated in the bio-ink [23]. Correspondingly, to arrive at optimal cell viability, we optimized conditions for skin 3D bioprinting by testing nozzles of different diameters to observe whether they pose a critical influence on the survival rate of the cells in the skin constructs. Specifically, we tested multiple nozzles the diameter of which was 0.33 mm, 0.41 mm and 0.51 mm, respectively, during the extrusion printing. After printing the cell-laden bio-ink with these different nozzles, the live/dead assay was carried out.

**Figure 2.**
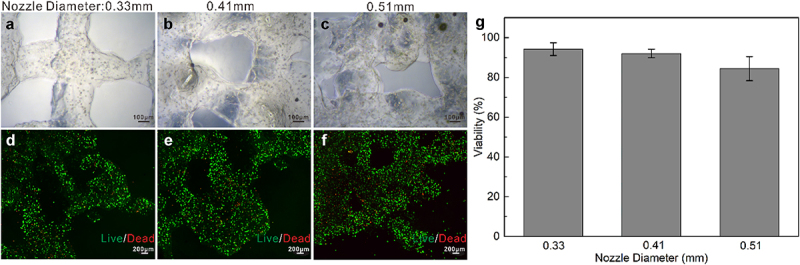
Optimization of printing conditions. A-C: Brightfield images of cellular constructs printed by the 0.33 mm, 0.41 mm, and 0.51 mm nozzle on day 1 after printing; D-F: Live/dead images of the encapsulated cells in the bio-ink on day 7 after printing; G: Cell viability analysis (Day 7).

Figure 2 illustrated representative images for printed cellular constructs and the fluorescent images (live/dead, calcein‐AM/PI) of the embedded cells within the bio-ink on day 7. On the one hand, it is obvious that, as the nozzle diameter increases, the printing accuracy decreases, with the 0.33 mm nozzle rendering the most elaborate construct (Figure 2a-c). On the other hand, as shown in Figure 2d-f, predominant green fluorescence evidenced the population of live cells and indicated that under conditions of 0.33 mm, 0.41 mm and 0.51 mm nozzles, even on day 7 after printing, the viabilities of cells embedded in the bio-ink were significantly high (~95%, 93% and 86% respectively). That is, the 0.33 mm nozzle can effectively improve printing accuracy without compromising cell survival. Based on the results, we selected the nozzle of 0.33 mm in diameter for fibroblasts-laden structure printing in the current study.

### 3.3 Generation of full-thickness skin constructs

The schematic diagram shown in Scheme 1 illustrates the preparation steps to obtain the full-thickness human skin. A four-step strategy was implemented: fabrication of dermal skin constructs, coating with laminin, seeding of keratinocytes and ALI culture via sterile wire mesh.

**Figure 3.**
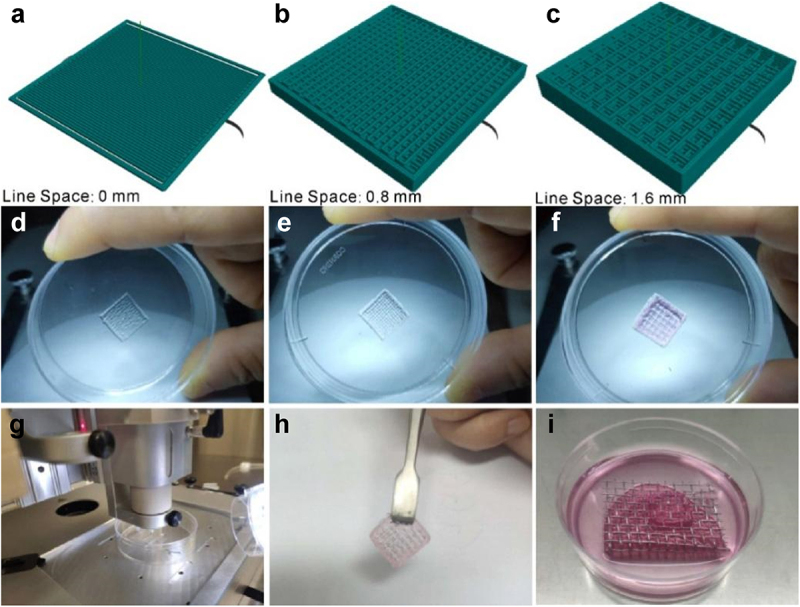
Fabrication of dermal layer constructs. A-C: different layers of the designed 3D dermal layer model; D-F: the corresponding printed dermal layer constructs; G: the printer in operation; H: the prepared dermal layer constructs; I: air-liquid interface culture of the full-thickness skin.

In the fabrication of dermal skin constructs, apart from the optimization of bio-ink and printing conditions, the elaborate design of the 3D skin model is equally of paramount importance. In this present study, as described in Materials and Methods, we devised a dermal skin model of 10 layers, wherein the initial 2 layers, the middle 4 layers and the top 4 layers were designed with interfilament distances of 0 mm, 0.8 mm and 1.6 mm, respectively. By doing so, on completion of the printing program, a groove-like pattern could be generated to accommodate the epidermal layers to be seeded for mimicking dermal-epidermal junctions of the human skin (Figure 3a-c).

As clearly demonstrated in Figure 3d-f, the printed dermal skin constructs matched well with the designed 3D model. After being gelled by 2% CaCl_2_, the printed dermal skin construct possessed a certain elasticity and toughness and thus can be readily taken out and transferred (Figure 3h). On top of this, in an effort to further enhance the junctions of the dermis and epidermis, laminin solution was plated on the bio-printed construct before the epidermal cells were seeded. Following this, as shown in Figure 3i, we used sterile wire mesh instead of Transwell here for the ALI culture of the full-thickness skin. Based on all the above steps, a full-thickness skin construct was thus generated.

### 3.4 Histological evaluation of full-thickness skin constructs

**Figure 4.**
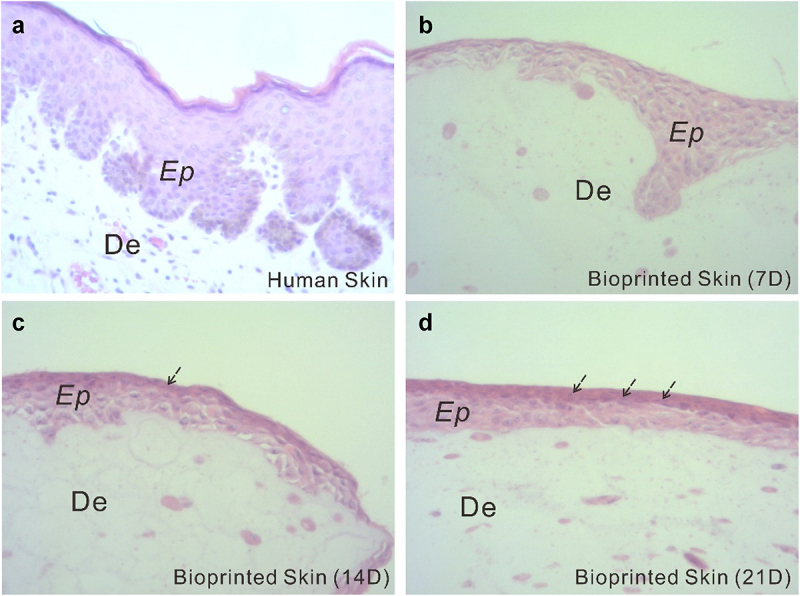
Histological and morphological characterization of the printed skin. Optical microscopy images of normal human skin (a) and printed skin after 7, 14, and 21 days of culture (b-d). Tissues were stained with H&E.

To assess the structural components of the fabricated full-thickness skin, histological analysis was performed. Figure 4 shows H&E staining of the full-thickness skin harvested after 7, 14, and 21 days of ALI culture, with a normal human skin serving as a control. As seen from the histological characterization, the printed skin was morphologically representative of the normal human skin. Specifically, in the printed full-thickness skin, the cells of the epidermal layer, when viewed from the bottom up, were more and more closely arranged and gradually flattened with the increase of culture duration, indicating a tendency to form cuticles. Apparently, the printed skin was capable of producing a dense, well-organized, and terminally differentiated epidermis.

### 3.5 Immunohistochemical evaluation of full-thickness skin constructs

As is well-known, correct stratification and localization of biomarkers of epidermal differentiation of the skin construct are crucial indicators for normal epidermal behavior [24]. Therefore, we further compared the full-thickness skin constructs with normal human skin in terms of biomarkers using immunohistochemistry.

**Figure 5.**
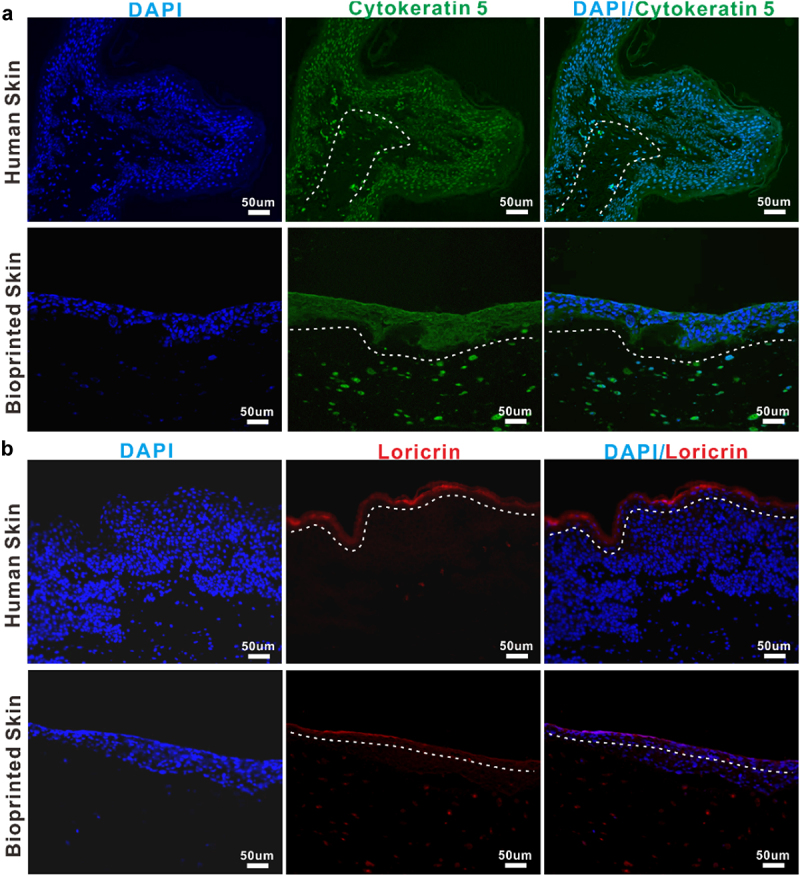
Immunohistochemical evaluation of full-thick skin constructs.

Immunohistochemical characterization shown in Figure 5 exhibited that the epidermis of printed skin was not only morphologically similar to that of the human skin but also expressed the specific maker related to epidermal differentiation and proliferation, such as cytokeratin 5 (Figure 5a). In addition, the skin barrier function, known to be closely related to the formation of the stratum corneum, was presented in the printed skin, primarily owing to the presence of a fully differentiated epidermis demonstrated by the expression of the specific maker loricrin (Figure 5b). Nevertheless, it can be found that the stratum corneum in the printed full-thickness skin was thinner than that in the normal human skin. In conclusion, these results suggested the formation of a well-differentiated but thin epidermis.

Representative images of immunofluorescence staining of human normal skin and bioprinted skin constructs. Printed skin showed positive expression of cytokeratin 5(A) and loricrin (B), similar to normal human skin. Cell nuclei were stained with DAPI (blue). The white dotted line indicates the epidermal-dermal boundary. Scale bar: 50 μm.

## 4. Discussion

Emerging as an advanced and promising technique evolved from conventional 3D printing, 3D bioprinting possesses the considerable potential to create highly mimicked tissue or organs with a high degree of repeatability and flexibility [25]. Over the past decade, 3D bioprinting has been extensively employed in fabricating biological tissues in various fields including bone, heart, cartilage, blood vessels, muscle, liver and skin, among others [12,26]. In terms of artificial skin or skin equivalents, in contrast to conventional skin tissue engineering methods [27,28], 3D bioprinting offers the potential to recapitulate the macroscale architectures and micro-features of the natural skin and enables the rapid and reliable production of biomimetic cellular skin substitutes owing to their unique ability to precisely pattern living cells in pre-defined spatial locations [24,29].

Typically, the 3D bioprinting process of human skin tissue can be summarized as the following three steps: 1) cell selection and cultivation, bio-ink selection and preparation, and 3D model design of skin; 2) the actual printing process and maturation of the printed skin constructs; and 3) histological evaluation and biochemical characterization of the printed skin tissue [30]. At present, extrusion-based bioprinting, inkjet bioprinting, and laser-assisted bioprinting are the most commonly used bioprinting techniques for the fabrication of skin tissue [24,29]. Here, we fabricated a full-thickness human skin model with success by using extrusion-based bioprinting as per the general process.

Bio-inks, as one of the most essential elements for successful 3D bioprinting, provide structural, mechanical and biological cues for the cells embedded therein, and thus ought to possess outstanding biological, rheological, and mechanical properties including excellent cell biocompatibility, high printing fidelity, favorable biodegradability and high mechanical strength [25]. Currently, a variety of hydrogel materials (such as collagen, gelatin, fibrin, chitosan and hyaluronic acid and/or combinations thereof) have been used as bio-inks in bioprinting skin tissues [31]. In this work, based on these previous reports and in consideration of the desirable properties, a brand-new bio-ink that contains 5% (w/v) gelatin, 1% alginate, and 2 mg/ml fibrinogen was selected through a great number of tests. Such a bio-ink brought about high cell viability and availed cell adhesion. Despite the superb performance of our bio-ink, bio-inks need to be further optimized with respect to biocompatibility and mechanical strength in future work. In this regard, the ColMa (methacrylated collagen)-based bio-ink is supposed to be an excellent choice.

It is generally recognized that during extrusion printing, shear stresses applied to the bio-ink through a fine diameter nozzle can potentially damage cells [23,32]. Typically, the finer the nozzle, the greater the shear pressure. However, in our study, inconsistent with the expectation, the highest shear pressure induced by using the finest 0.33 mm nozzle failed to pose the most harmful impact on the encapsulated cells. Moreover, it is conjectured that in the case of the 0.51 mm nozzle, the appearance of slightly more dead cells in the middle of interfilament could be attributed to the insufficient nutrient and waste diffusion.

In addition to cell and bio-ink selections, 3D model design of skin is crucial prior to the actual 3D printing process [23,29,32]. In this connection, our groove-like pattern resulted from the ingenious design of the dermal skin model with different layers having different interfilament distances indeed does favor to the tight dermal-epidermal junctions in the later culture. Besides, it is also worth mentioning here that the sterile wire mesh used in the ALI culture of the full-thickness skin is pretty economic, easily available, compatible with any size dish and can be added with a medium of desired volume to prevent nutrient and oxygen deficiency, thereby enormously saving the cost and simplifying the operation.

Histological evaluation of the printed skin confirmed the formation of a well-developed epidermis within the construct; however, a subtle difference in the thickness of epidermal layer definitely occurred between the printed skin and normal human skin. In accordance with the histological analysis, the positive expression of critical biomarkers (cytokeratin 5 and loricrin) tightly associated with epidermal differentiation and stratum corneum formation shown in immunohistochemical detection indicated the presence of a fully differentiated but thin epidermis. We speculate that such a thin epidermal layer in our printed skin is most likely caused by the fact that during the post-printing culture, non-optical culture conditions were utilized. In the follow-up work, we will cultivate the printed skin construct under optical culture conditions. Plus, we will optimize the cytokines mix to promote the formation of full-thickness skin mimicking human skin. Moreover, fewer cell types, less extracellular matrix and less cell density in our fabricated full-thickness skin relative to the authentic human skin are all the possible causes of the thin epidermis, and thus need to be improved. In the future study, the epidermal thickness could be also improved by means of prolonging the culture duration and optimizing the medium components.

Taken together, in spite of the successful bioprinting of full-thickness human skin tissues, to recapitulate deeper complexity of native human skin, further optimization is required, including incorporation of other skin-related cell types such as melanocytes, endothelial cells, adipocytes, Langerhans cells, hair follicles, sweat glands and stem cells; development of novel bio-inks with better performance; and improvement of bioprinting conditions such as the concentration of bio-ink, temperature of the nozzle and forming space.

## 5. Conclusions

In this study, we first fabricated dermal skin constructs through extrusion-based bio-printing. High cell viability within the printed constructs was maintained for at least 7 days after printing. The incorporation of laminin solution and the seeding of epidermal cells followed by ALI culture enabled the fabrication of full-thickness skin. Furthermore, the histological evaluation and immunochemical analysis revealed that the full-thickness skin presented here closely resembled native human skin, both morphologically and biologically. In a nutshell, this study provides a simple, robust and cost-saving fabrication of full-thickness skin constructs, which can be applied for drug screening, cosmetic testing and clinical treatment.

## Highlights

- A novel bio-ink composed of gelatin, sodium alginate and fibrinogen was provided in this article and laminin was added to facilitate the formation of the epidermis.
- The ALI culture was performed by virtue of sterile wire mesh, which possesses the advantages of saving the cost and simplifying the operation..
- The bio-printed skin was not only morphologically representative of the human skin, but also expressed the specific markers related to epidermal differentiation and stratum corneum formation.

## References

[1] Frueh FS, Menger MD, Lindenblatt N, et al. Current and emerging vascularization strategies in skin tissue engineering. Crit Rev Biotechnol. 2017;37(5):613–625. 27439727 10.1080/07388551.2016.1209157

[2] Miguel SP, Cabral CSD, Moreira AF, et al. Production and characterization of a novel asymmetric 3D printed construct aimed for skin tissue regeneration. Colloids Surf B Biointerfaces. 2019;181:994–1003. 31382351 10.1016/j.colsurfb.2019.06.063

[3] Shpichka A, Butnaru D, Bezrukov EA, et al. Skin tissue regeneration for burn injury. Stem Cell Res Ther. 2019;10:94. 30876456 10.1186/s13287-019-1203-3 PMC6419807

[4] Gzik-Zroska B, Joszko K, Wolański W, et al. Assessment of the impact of decellularization methods on mechanical properties of biocomposites used as skin substitute. Materials. 2021;14:4785. 34500876 10.3390/ma14174785 PMC8432536

[5] Hakimi N, Cheng R, Leng L, et al. Handheld skin printer: in situ formation of planar biomaterials and tissues. Lab Chip. 2018;18:1440–1451. 29662977 10.1039/c7lc01236e PMC5965293

[6] Brockmann I, Ehrenpfordt J, Sturmheit T, et al. Skin-derived stem cells for wound treatment using cultured epidermal autografts: clinical applications and challenges. Stem Cells Int. 2018;2018:4623615. 29765411 10.1155/2018/4623615 PMC5889868

[7] Almeida A, Sarmento B, Rodrigues F. Insights on in vitro models for safety and toxicity assessment of cosmetic ingredients. Int J Pharm. 2017;519:178–185. 28104405 10.1016/j.ijpharm.2017.01.024

[8] Pfuhler S, Kirst A, Aardema M, et al. A tiered approach to the use of alternatives to animal testing for the safety assessment of cosmetics: genotoxicity. A COLIPA analysis. Regul Toxicol Pharmacol. 2010;57(2–3):315–324. 20382194 10.1016/j.yrtph.2010.03.012

[9] Matai I, Kaur G, Seyedsalehi A, et al. Progress in 3D bioprinting technology for tissue/organ regenerative engineering. Biomaterials. 2020;226:119536. 31648135 10.1016/j.biomaterials.2019.119536

[10] Gomez-Blanco JC, Galvan-Chacon V, Patrocinio D, et al. Improving cell viability and velocity in mu-extrusion bioprinting with a novel pre-incubator bioprinter and a standard FDM 3D printing nozzle. Materials (Basel). 2021;15(1):14. 34198815 10.3390/ma14113100 PMC8201198

[11] Murphy SV, Atala A. 3D bioprinting of tissues and organs. Nat Biotechnol. 2014;32(8):773–785. 25093879 10.1038/nbt.2958

[12] Mandrycky C, Wang Z, Kim K, et al. 3D bioprinting for engineering complex tissues. Biotechnol Adv. 2016;34:422–434. 26724184 10.1016/j.biotechadv.2015.12.011 PMC4879088

[13] Pahlevanzadeh F, Mokhtari H, Bakhsheshi-Rad HR, et al. Recent trends in three-dimensional bioinks based on alginate for biomedical applications. Materials (Basel). 2020;13 (18):3980. 10.3390/ma13183980 PMC7557490 32911867

[14] Lee V, Singh G, Trasatti JP, et al. Design and fabrication of human skin by three-dimensional bioprinting. Tissue Eng Part C Meth. 2014;20:473–484. 10.1089/ten.tec.2013.0335 PMC4024844 24188635

[15] Admane P, Gupta AC, Jois P, et al. Direct 3D bioprinted full-thickness skin constructs recapitulate regulatory signaling pathways and physiology of human skin. Bioprinting. 2019;15:e00051.

[16] Cubo N, Garcia M, Del Canizo JF, et al. 3D bioprinting of functional human skin: production and in vivo analysis. Biofabrication. 2016;9:015006. 27917823 10.1088/1758-5090/9/1/015006

[17] Liu J, Li C, Cheng S, et al. Large‐scale high‐density culture of hepatocytes in a liver microsystem with mimicked sinusoid blood flow. J Tissue Eng Regen Med. 2018;12:2266–2276. 30350403 10.1002/term.2758

[18] Kim BS, Kwon YW, Kong JS, et al. 3D cell printing of in vitro stabilized skin model and in vivo pre-vascularized skin patch using tissue-specific extracellular matrix bioink: a step towards advanced skin tissue engineering. Biomaterials. 2018;168:38–53. 29614431 10.1016/j.biomaterials.2018.03.040

[19] Fortunato GM, De Maria C, Eglin D, et al. An ink-jet printed electrical stimulation platform for muscle tissue regeneration. Bioprinting. 2018;11:e00035.

[20] Yang H, Sun L, Pang Y, et al. Three-dimensional bioprinted hepatorganoids prolong survival of mice with liver failure. Gut. 2021;70:567–574. 32434830 10.1136/gutjnl-2019-319960 PMC7873413

[21] de Melo BAG, Jodat YA, Cruz EM, et al. Strategies to use fibrinogen as bioink for 3D bioprinting fibrin-based soft and hard tissues. Acta Biomater. 2020;117:60–76. 32949823 10.1016/j.actbio.2020.09.024

[22] Laurens N, Koolwijk P, de Maat MP. Fibrin structure and wound healing. J Thromb Haemost. 2006;4:932–939. 16689737 10.1111/j.1538-7836.2006.01861.x

[23] Aguado BA, Mulyasasmita W, Su J, et al. Improving viability of stem cells during syringe needle flow through the design of hydrogel cell carriers. Tissue Eng Part A. 2012;18(7–8):806–815. 22011213 10.1089/ten.tea.2011.0391 PMC3313609

[24] Yan WC, Davoodi P, Vijayavenkataraman S, et al. 3D bioprinting of skin tissue: from pre-processing to final product evaluation. Adv Drug Deliv Rev. 2018;132:270–295. 30055210 10.1016/j.addr.2018.07.016

[25] Heinrich MA, Liu W, Jimenez A, et al. 3D Bioprinting: from Benches to Translational Applications. Small. 2019;15:e1805510. 31033203 10.1002/smll.201805510 PMC6752725

[26] Huang Y, Zhang XF, Gao G, et al. 3D bioprinting and the current applications in tissue engineering. Biotechnol J. 2017;12(8). 10.1002/biot.201600734 28675678

[27] Wessels Q. Engineered alternative skin for partial and full-thickness burns. Bioengineered. 2014;5(3):161–164. 24651001 10.4161/bioe.28598 PMC4101007

[28] Carretero M, Guerrero-Aspizua S, Del Rio M. Applicability of bioengineered human skin: from preclinical skin humanized mouse models to clinical regenerative therapies. Bioengineered Bugs. 2014;2:203–207. 10.4161/bbug.2.4.16112 21829094

[29] Weng T, Zhang W, Xia Y, et al. 3D bioprinting for skin tissue engineering: current status and perspectives. J Tissue Eng. 2021;12:20417314211028574. 34345398 10.1177/20417314211028574 PMC8283073

[30] Gao C, Lu C, Jian Z, et al. 3D bioprinting for fabricating artificial skin tissue. Colloids Surf B Biointerfaces. 2021;208:112041. 34425531 10.1016/j.colsurfb.2021.112041

[31] Xu J, Zheng S, Hu X, et al. Advances in the research of bioinks based on natural collagen, polysaccharide and their derivatives for skin 3D bioprinting. Polymers. 2020;12(6):1237. 10.3390/polym12061237 PMC7362214 32485901

[32] Kim W, Kim G. 3D bioprinting of functional cell-laden bioinks and its application for cell-alignment and maturation. Appl Mater Today. 2020;19:100588.

